# 

**Published:** 1909-03-15

**Authors:** 


### ANNOUNCEMENTS.

---

INTERSTATE DENTAL FRATERNITY.—The annual Meeting of the Interstate Dental Fraternity of the United States and Canada, will be held at Birmingham, Ala., during the meeting of the National Dental Association. The meeting will be in charge of Dr. R. H. Welsh, Secretary of the I. D. A. for Louisiana.

R. M. SANGER, *National Secretary.*

---

ILLINOIS STATE DENTAL SOCIETY.—The forty-fifth annual meeting of the Illinois State Dental Society will be held at Danville, May 11th, 12th, 13th and 14th, 1909.

R. J. HOOD, *Secretary,*
Sparta, Illinois.

---

EASTERN KENTUCKY DENTAL ASSOCIATION.—This dental society has just been organized and will hold its next meeting at Ashland, Ky., Saturday, April 3rd, 1909. The meetings will be held every three months at such places as the society may select. The officers are: President, Dr. R. H. Leete, of Prestonsburg; Vice President, Dr. J. A. Louber, Catlettsburg; Secretary, Dr. P. H. Williams, Ashland.

---

The 39th Annual Convention of the Kentucky State Dental Association will convene at Crab Orchard Springs, Kentucky, May 17th, 18th and 19th, 1909. We anticipate a most interesting and profitable meeting at this most popular central Kentucky resort. A cordial invitation is extended to all ethical members, of the profession.

W. M. RANDALL, *Secretary.*

---

VIRGINIA STATE DENTAL ASSOCIATION.—The 40th Annual Session of the Virginia State Dental Association will be held at the Mechlenburg, Chase City, Va., July 21st, 22nd and 23rd, 1909. Every effort is being made to make this the most interesting and successful meeting of our Society. Men of national reputation will give clinics and read papers. All ethical practitioners are cordially invited to attend.

W. H. PEARSON, *Cor. Secretary.*

---

MISSOURI STATE DENTAL ASSOCIATION.—The 44th annual meeting of the Missouri State Dental Association will convene at Kansas City, Missouri, May 26th, 27th and 28th, 1909. A good live program is in course of preparation.

Respectfully,

J. F. WALLACE, *Cor. Secretary.*

---

INTERNATIONAL DENTAL CONGRESS.—Berlin 23.28th August 1909. The congress will take place in the Reichstag Building. The Honorary President of the congress is Geheimrat Prof. Dr. Waldeyer, Director of the 1st Anatomical Institute. Honorary members: Dr. Naumann, Chief of the Medical Department of the "Kultusministerium." Geheimrat Prof. Dr. Kirchner. The business of the congress is conducted by the following committees: 1. Committee on Organization. 2. Berlin Local Committee. 3

Chairmen of the different Sections. I. The Committee on Organization consists of 15 gentlemen. President: Privy Councillor Professor Dr. Walkoff, Munchen, Briennerstr. 47. Vice Presidents: Professor Dieck. M, D., Berlin, Potsdamerstr. 113. Professor Hahl, Berlin, Lutzowstr.53. Hielscher, Coln o. Rh., Hohenzollernring 30. Secretary General: Schaeffer-Stuckert, D.D.S., Frankfort a. M., Kettenhofweg 29.; Secretary:Konrad Cohn, M.D., Berlin, Potsdamerstr. 46.; Treasurer: Blume, Berlin W., Unter den Linden 41; II. The Berlin Local Committee is composed of 38 gentlemen. Presidents: Professor Guttmann, court dentist, Potsdam; Robert Richter, D.D.S., Berlin, Victoriastr,23; Dr. P. Ritter, Berlin, Koniggratzerstr,94; Secretaries: Wiedemann, Berlin, Bulowstr, 1; Gutmann, Berlin, Alexanderstr ,71; Pursche, Berlin, Rankestr,30; Treasurer: Helm, Charlottenburg, Berlinerstr, 169a; The Berlin Local Committee will arrange the order of proceedings, will be pleased to procure lodgings for foreign colleagues and supply them with all information concerning their journey, their stay in Berlin etc. All questions regarding the above mentioned subjects should be addressed to the president of the local committees, Professor Guttmann, Potsdam. III. The Chairmen of the Sections. 12 sections have been formed, all of which can hold in sessions in the Reichstag Building simultaneously. Section I. Anatomy, Physiology, Histology. Chairman: Dr. Adloff in Konigsberg i. Pr. Weissgerberstr, 6.7. Section II. Pathology,Bacteriology. Chairman: Professor Dr. Romer, Strassburg i. E. Section III. Chemistry, Physics,Metallurgy. Chairman: C. Birgfeld, Hamburg, Alsterdamm 1. Section IV. Diagnosis and special Therapeutics, Materia Medica. Chairman: Prof. Dr. Michel, Wurzburg. Section V. Surgery of the Mouth and surgical Prothesis. Chairmen Geheimrat Prof. Dr. Partsch, Breslau, Prof. Dr. Schroder Berlin. Section VI. General and Local Anaesthesia. Chairman: University Lecturer Dr. Fischer,Griefswald. Section VII. Preservative Dentistry. Chairman: Prof.Dr.

Sachs, Berlin, Kurfurstendamm 247. Section VIII. Artificial teeth incl. crown-and bridge-work, Ceramic. Chairman: Prof. Dr. Riegner, Breslau. Section IX. Orthodonty, Chairman, Heydenhauss M.D., Berlin, Potsdamerstr. 121. Section X. Hygiene of the Mouth and Teeth. Chairman: Dr. C. Rose, Dresden. Section XI. Instruction and Legislation. Chairman: Dr. Ritter, Berlin, Koniggratzerstr. 94. Section XII: History and Literature. Chairman: Hoffendahl, Berlin, Schoneberger Ufer 20. During the week of the congress an official daily journal will be published in 3 languages (German, English, French). Editor: Konrad Cohn M.D., Berlin, Potsdamer Strasse 46. All gentlemen desiring to give papers or demonstrations, are requested to announce the same as early as possible to the chairman of the respective section. An international scientific and industrial exhibition will be combined with the congress. Professor Dr. Dieck, Berlin, Potsdamerstr. 113, Villa 3, has taken charge of the management of this exhibition, which is to be conducted on a large scale, and will furnish further information regarding the same. A meeting of American dentists who wish to attend this congress or participate has been called at the time of the National Dental Association meeting in Birmingham, Alabama, March 31st to April 1st. Dr. Burton L. Thorpe, 3605 Lindell Blv., St, Louis, Mo., is Secretary of the American Committee and will give all information necessary to any one desiring to participate At the last meeting of the committee on Organization it was decided that the fee for membership be fixed at 25 marks ($6..00), which sum will also entitle the holders of membership cards to a copy of the Transactions when published. For participation in the social functions additional cards will be issued by the Berlin Local Committee at a very low price. A guarantee fund of 20,600 marks has already been subscribed, and it has been decided not to call upon foreign visitors for financial or administrative support. A hearty invitation is extended to all American *confreres*.

PROGRAM.

The following provisional program has been arranged:

*SUNDAY, August* 22d.—Meeting of the Federation Dentaire Internationale. Evening: Reception of the guests at the Reichstagsgebaude.

*MONDAY, August* 23d.—Morning: Opening session. After the official addresses of welcome, four orators (German, English, French and American) will speak on subjects chosen by themselves and important for the entire profession. The National Committees of the respective countries have each been requested to nominate their orator. Evening: Reception given by the city of Berlin at City Hall.

*TUESDAY, August* 24th.—9 A. M. to 2 P. M.: Sessions of the Sections. Evening: Banquet in the halls of the Zoological Gardens.

*WEDNESDAY, August* 25th.—9 A. M. to 2 P. M.: Sessions of the Sections. Evening: Fiftieth anniversary of the Central Verein Deutscher Zahnarzte (Central Association of German Dentists) in the halls of the Rheingold.

*THURSDAY, August* 26th.—Second general session in the great hall of the Reichstagsgebaude. Subjects and questions will be discussed by speakers appointed by the different countries. Evening: at the disposal of the congressists.

*FRIDAY, August* 27th.—9 A. M. to 2 P. M.: Sessions of the Sections. Evening: Reception in honor of the congressists given by the *confreres* of Berlin and of the province of Brandenburg. Special train to Wannsee.

*SATURDAY, August* 28th.—9 A. M. to 12 M.: Sessions of the Sections (passing of resolutions) and meeting of the

Federation Dentaire Internationale. 3 P. M. Closing sessions. Acceptance of the resolutions of the Congress. Evening: Farewell banquet at the Halensee Terraces. On Sunday and after, groups of the congressists will visit German cities and universities. The Bureau of the congress will be opened four weeks before the opening of the congress. A postal, telegraph, and telephone office will be established, also refreshment rooms. The size of the Reichstagsgebaude will render it possible for the different sections to meet simultaneously, so that the participants may hear lectures in different sections on one day. In order to facilitate conversation between men of different nationalities, those *confreres* who speak English will wear a blue badge, those who speak French a red badge. The Hamburg-American Packet Co. allows to members of the congress a reduction of 25 per cent. except during the height of the season. The Berlin Local Committee will be pleased to procure lodgings for foreign colleagues and supply them with all the information concerning their journey, their sojourn in Berlin, etc. The prices of rooms in hotels vary from 2..50 to 30 marks per day ($0..60 to $7..00. All questions regarding this subject should be addressed to the president of the Local Committee, Professor Guttman, Postdam. In order to make the visitors acquainted with the sights of Berlin and its environs, ably conducted excursions have been arranged for. The scientific institutions of importance will also be open to visitors.

---

THE SEVENTH DISTRICT DENTAL SOCIETY of the State of New York will hold its Annual Meeting in Rochester, N. Y., on the 16th and 17th of April at the Seneca Hotel.

Plans are under way to make this the largest and most attractive meeting in various points of interest, which has ever been held in New York State, and all dentists within a radius of three hundred miles will be greatly benefited by coming.

There will be a large number of clinics at the chair and also table clinics, and several interesting papers will be read.

The manufacturers of dental instruments and supplies will also aid in making this a large meeting, and a most cordial invitation is extended to all who will come.

Business Committee,
LEWIS S. GOBLE, *Chairman*,
EDWARD L. SCHLOTTMAN,
CLARENCE A. THORN,
CLINT W. LA SALLE, *Secretary*,
Rochester, N. Y.

---

NORTHERN OHIO DENTAL ASSOCIATION.—This society will hold its next meeting at Cleveland, June 1st, 2nd and 3rd, in the Central Y. M. C. A. Building. An elaborate program is being provided.

DR. D. H. ZIEGLER, *President*.

